# Emotional Intelligence and Prosocial Behavior in College Students: A Moderated Mediation Analysis

**DOI:** 10.3389/fpsyg.2021.713227

**Published:** 2021-09-06

**Authors:** Haiying Wang, Shuang Wu, Weichen Wang, Chao Wei

**Affiliations:** School of Psychology, Northeast Normal University, Changchun, China

**Keywords:** emotional intelligence, prosocial behavior, social support, self-esteem, moderated mediation

## Abstract

This study examined the relationship between emotional intelligence (EI) and prosocial behavior (PSB) and constructed a model for their interaction by examining the mediating effect of social support (SS) and the moderating effect of self-esteem (SE) in this relationship. A total of 742 college students aged from 18 to 20 in Northeast China (*M*_age_ =19.42 ± 0.53 years) completed a survey measuring the Emotional Intelligence Scale, Prosocial Tendencies Measurement Scale—Chinese Version, Perceived Social Support Scale, and Self-Esteem Scale. The results showed that: (1) EI positively predicted PSB; (2) SS partially mediated the relationship between EI and PSB; and (3) SE moderated the direct effect of EI on PSB and the relationship between SS and PSB. That is, when the SE of college students was higher, the effect of SS in promoting PSB was enhanced. Therefore, our results suggested that under the influence of both internal and external factors, there is an indirect effect of EI on PSB. This finding may potentially provide a theoretical basis for designing college students' mental health courses and cultivating PSB in college.

## Introduction

Prosocial behavior (PSB) refers to all behaviors that are favorable to others and conducive to social harmony, such as helping, cooperating, sharing, and comforting (Eisenberg et al., 2006). For the individual, PSB can promote positive social adaptation, which is an important indicator of individual socialization development; for society, PSB can help people maintain a good relationship with each other, which is conducive to justice, harmony, and the development of the entire society (Penner et al., 2005; Wittek and Bekkers, 2015; Ding and Lu, 2016; Ding et al., 2016). PSB not only benefits others and society but also has a positive role in promoting the mental health of those who engage in it and those who receive it, as well as the development of human society (Kou et al., 2007; Yang et al., 2016; El-Khodary and Samara, 2019; Aycock et al., 2020). College students are the major workforce in China. Although the PSBs and its tendencies that college students exhibited in social activities are of great significance, the current situation is not optimistic. Some results showed that college students are lack a sense of security in real life, far away from social groups, self-centered and lack a sense of responsibility for PSB. Therefore, when they faced situations requiring helps, they are willing to have PSB but the duration is relatively short (Xia and Li, 2016; Xiu, 2018). Since urging people to have more PSB can cultivate and develop positive attitude and build a harmonious and stable society, the cause of PSB and the ways to promote individuals to have more PSBs are also worth studying in psychology. In recent years, numerous studies have examined the factors influencing PSB (Ruan, 2014; Dong et al., 2015; Ding et al., 2016; Zhao et al., 2020; Serrano-Montilla et al., 2021). Studies have examined the two main factors affecting PSB, which are external social factors and internal individual factors (Xiao et al., 2014). So far, most studies have focused on the effects of external macro social factors (Wentzel et al., 2007; Yuan et al., 2019) and individual factors (Eisenberg et al., 2010; Liu et al., 2020) on PSB separately, and little research has been done to examine the interaction effect of internal and external factors on PSB. PSB plays an important role in the socialization of college students, therefore, a deep exploration of the joint effect of internal and external factors of PSB in college students is called for.

Emotional intelligence (EI) is the ability of individuals to monitor their own and others' emotions, and to identify and use this information to guide their thoughts and behaviors (Salovey and Mayer, 1989). According to Eisenberg's PSB theory, the process by which individuals produce PSB includes three stages: paying attention to the needs of others, determining an intention to help others, and linking intention and behavior (Yang et al., 2017). Vorbach and Foster (2002) studied the relationship between emotional components (identifying others' emotions, emotional regulation) and social components (relationship quality and PSB) and found that the ability to identify others' emotions is correlated positively with PSB but negatively with aggressive behavior. In the need-awareness stage of PSB, the individual pays attention to whether others need help and this involves the perception and evaluation of the emotional perception and expression ability of EI on the environment of others and the emotions of others. Simultaneously, after determining that the other person needs help, the individual needs to choose whether to help the seeker. At this time, the understanding and management dimensions of EI are called on so the individual can organize and analyze the information they have and assess whether their intentions to engage in PSB are in line with the current situation (Xu and Li, 2020). Of course, the emotional management dimension of EI also plays a significant role in the final stage of connection between intentions and behavior (Glazer, 2021). Thus, in the process of PSB production, EI plays an important role. The higher a person's EI, the stronger their emotional perceptions of others will be, and the higher the probability that they will engage in PSB.

The ability to perceive and appraise others' emotions may provide information relevant to PSB. Studies have shown that an individual's EI is significantly positively correlated with PSB (Marc et al., 2004; Martí-Vilar et al., 2019). Mayer and colleagues also found that individuals with high EI engage in more positive social behaviors (Mayer et al., 2004). For instance, individuals who perceive others' levels of fear accurately also demonstrate more PSB in social interactions (Kaltwasser et al., 2016). Charbonneau and Nicol (2002) found that EI is positively correlated with good social relations and has a significant predictive effect on PSB. Individuals with high EI show more PSB, better empathy, and fewer negative behaviors in interactions with peers (Ciarrochi et al., 2002; Mavroveli and Sánchez-Ruiz, 2011). In primary school, EI contributes to the socialization. Poulou (2010) conducted a survey on adolescents aged from 12 to 14 and found that students with high EI and better social skills are more likely to exhibit PSB. Recent findings indicated that EI facilitates PSB in adults (Kaltwasser et al., 2016; Martin-Raugh et al., 2016). Furthermore, emotional understanding can significantly and positively predict prosocial tendencies (Liu and Zou, 2010). Although most studies have confirmed the relationship between EI and PSB, the potential mechanism by which EI affects PSB is not clear. According to previous studies, a direct or indirect relationship between EI and PSB may exist under given conditions or be moderated by some factors. Therefore, the mediating and moderating role of EI on PSB needs to be further explored in order to cultivate individual's PSB and provide method and basis for designing college students' mental health courses. Integrating these findings, we postulate the following: Hypothesis 1: EI is positively associated with PSB.

Social support (SS) refers to types of psychological help or material support such as care, respect, and meeting needs from family members, friends, organizations, and other members of society (Feng et al., 2018; Yao et al., 2018). It is an important social resource which is an individual-centered system composed of social interactions between individual and people around them (Zhu et al., 2016). SS is an important personal resource and plays an important role in maintaining and promoting physical and mental health. According to the ability model of EI (Mayer et al., 1999) and mixed model of EI (Goleman, 1995), SS is closely related to EI and PSB.

On the one hand, EI can predict individual SS. The ability model of EI proposed by Mayer et al. (1999) and the mixed model of EI proposed by Goleman (1995) all illustrate the proposition that individuals with high EI can effectively identify and express their emotions; understand the feelings of others; and establish and maintain mutually satisfactory and responsible interpersonal relationships with them (Bar-On, 2005). At the same time, they can establish a stable connection with the outside world and obtain more SS. Some studies have found that EI is a key factor in cultivating communication skills (Cheng and Zou, 2011) and that the individual with higher EI have better interpersonal relationships (Tang et al., 2015). EI is significantly positively correlated with SS (Kong et al., 2012; Xiao and Hou, 2017; He et al., 2020). In particular, individuals with high EI are more active in interpersonal relationships (Schutte et al., 2001), and receive more emotional support from social support system, when they faced bad emotions, they will seek helps from the system (Salovey et al., 2002). Moreover, He et al. (2020) found that SS plays a part of mediating role between EI and PSB. In other words, the higher level of EI an individual has, the more SS one receives, and it has more significant impact on one's mental health. Ma and Wang (2013) also found that college students with high EI have a strong ability to identify and judge their own emotions and those of others, making them more likely to have a large number of high-quality social networks, which is conducive to their obtaining better external SS.

On the other hand, individuals with more SS tend to engage in more PSB (Ciarrochi et al., 2001). PSB occurs in the process of communication, and experience can influence the occurrence of PSB (Lawler and Thye, 1999; Cirelli et al., 2014). Studies have proven that SS is positively correlated with PSB in college students (Tian et al., 2016; Guo, 2018; Wouter et al., 2018; Li et al., 2019). Wang (2011) explored the characteristics of PSB and the relationship between SS and PSB thoroughly and found that PSB is affected by multiple factors, such as SS, individual satisfaction, and teacher engagement. When individuals feel they have a good interpersonal environment and close organizational relationships, they will have a strong sense of belonging, which promotes altruistic behavior (Twenge et al., 2007; Wei et al., 2017). Positive SS provides a good environment for the practice and development of PSB (Guzman et al., 2013).

In summary, this study investigated that whether there are important relationships among EI, PSB and SS. Zhao et al. (2020) conducted a three-wave longitude study of adolescents to explore the effects of EI on positive and negative emotions, in which SS and PSB as mediation variables affect adolescents' emotions. The results indicate that there is a positive correlation among them. But so far, there is lack of a test that SS may play a mediating role between EI and PSB. Therefore, we postulate the following: Hypothesis 2: SS plays a mediating role to affect the relationship between EI and PSB.

Among the Big Five personality traits, agreeableness, conscientiousness, and neuroticism are highly correlated with PSB and can positively predict PSB (Ashton et al., 1998). According to Eisenberg's PSB theory, SE is a personality factor that motivates altruism and influences intentions to be helpful. The SE level of individuals is highly related to the occurrence of PSB (Qi and Liu, 2013; Wu et al., 2014; Qiao and Wu, 2016). Individuals who have higher SE will have a strong sense of self-worth and tend to be less worried about being threatened. Therefore, they will not be too immersed in self-focus but will devote positive attention to others and can be sensitive to subtle clues to others' needs (Liu et al., 2016). As Turowska (2010) proposed, SE as a personality tendency plays an extremely important role in the relationships between individuals. For example, individuals' altruistic behavior and tendency to cooperate are all related to the level of individual SE. Individuals with high SE have a better adaptive function; in turn, they are more willing to provide help to others (Butler and Gasson, 2005). EI is the ability to perceive emotions and use this information to guide one's behavior; its influence on PSB may be affected by SE. Meng et al. (2021) studied the relationship between SE and PSB and they found that individuals with higher SE were able to produce more empathy, which in turn affected the occurrence of PSB, that is, individuals with higher SE pay more attention to outside and have more emotional perception which is a dimension in the ability model of EI (Mayer et al., 1999). Ding and Ma (2013) found that college students with high SE pay more attention to others' evaluation of them and are good at restraining themselves. They also found it easier to manage their emotions and tend to have a positive attitude toward things. However, individuals with low SE have little self-affirmation, which can easily result in an inferiority complex and negative state, making it difficult for them to manage their emotions reasonably and leading to an unwillingness to engage in PSB. Integrating these findings, we postulate the following: Hypothesis 3a: SE plays a moderated role between EI and PSB.

There is a significant positive correlation between SE and SS (Peng et al., 2003). Based on Rosenberg's Social-Bonding Theory, low SE weakens social connection and thus reduces the consistency between individuals and social norms, thereby increasing aggression (Xin et al., 2007). Individuals with high EI rate themselves more objectively and positively, exhibit more confidence externally (Murrell et al., 2003) and therefore they use less aggressive or hostile behaviors to maintain SE (Li, 2016). The research of Wang and Wang (2005) also supported the above conclusion and indicated that SE is an important personality factor affecting individual PSB. Moreover, individuals with low SE tend to be fearful and have negative and pessimistic evaluations of themselves. In social life, they mostly give people the impression that they wish to dodge social interactions; they tend to come into contact with fewer people and to have a low probability of engaging in PSB (Shi et al., 2017). Thus, SE affects SS, and individuals with high SE tend to process information positively, whereas individuals with low SE are more likely to indulge in negative emotions and engage in negative behavior (Kernis, 2003). In this process, high-SE groups can be more sensitive to changes in their surroundings and tend to be willing to help others when they need it (Hou, 1990). Integrating these findings, we postulate the following: Hypothesis 3b: SE moderates the relationship between EI and PSB and the relationship between SS and PSB.

**Figure 1:**
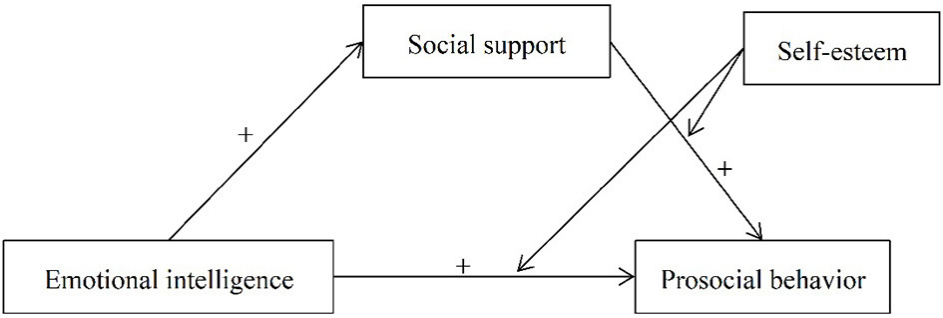
The moderated mediation effect among emotional intelligence, prosocial behavior, social support, and self-esteem. The + sign denotes a positive relationship for a pathway.

Although there is substantial evidence supporting a link between EI and PSB, the mechanisms underlying this link have not been extensively explored. The current study aimed to test an integrated moderated mediation effect to better understand the association between EI and PSB in college students. The first part of the effect examines whether SS mediates the association between EI and PSB. The second part of the effect includes SE as a moderator to understand whether it influences this association; it is hypothesized that SS and SE interact to determine PSB. Moreover, if SE moderates the association between SS and PSB, it is also likely that SE will conditionally influence the strength of the indirect association between EI and PSB. Based on previous findings (Wang and Wang, 2005; Turowska, 2010), SS would mediate the indirect effect when SE level was high, but the indirect effect might be small when SE is low. Thus, in this study, we posit the following moderated mediation effect (see Figure 1).

## Method

### Participants

A total of 780 college students were recruited from universities in northeast China by random sampling. Due to missing or invalid responses, 38 participants were not included in the analyses. Therefore, the final sample consisted of 742 participants (403 females, 54.3%), with the age from 18 to 20 (M = 19.42, SD = 0.53). In the final sample, there were 297 freshmen (40.0%), 143 sophomores (19.3%), 208 juniors (28.0%), and 94 seniors (12.7%). The participants all had normal visual acuity and no mental illness. The study was approved by the Academic Ethics Committee of the College of Psychology of Northeast Normal University.

### Measures

#### Emotional Intelligence

EI was assessed by the Emotional Intelligence Scale (EIS), developed by Salovey and Mayer, translated and revised by Wang (2002). It comprises 33 items, and items 5, 28, and 33 were scored in reverse (e.g., “I think it is difficult for me to understand the body language of others”). It includes four dimensions: emotional perception (12 items, e.g., “I understand the thoughts and feelings of others”), self-regulation of emotions (eight items, e.g., “I can control my emotions”), regulation of others' emotions (six items, e.g., “When others do well in a certain area, I will praise them”), and use of emotions (seven items, e.g., “When I feel a change in mood, some new ideas will spring up”). Participants rated the items on a 5-point Likert-type scale (1 = strongly inconsistent; 5 = strongly consistent), with higher total scores indicating that the more positive emotions an individual usually shows, the more impulsivity they can control, and the more clearly they express their feelings. Higher total scores also indicate that the individual has strong psychological resilience and high self-healing ability. The Cronbach's alpha was 0.904.

#### Prosocial Behavior

The Prosocial Tendencies Measurement Scale—Chinese Version (PTM) was used to assess PSB in the participating college students (Wei et al., 2017). The PTM consists of 23 items, which are all scored in forward and categorized into six dimensions: openness (four items, e.g., “I will try my best to help others under the eyes of public”), anonymity (five items, e.g., “I prefer to donate anonymously”), altruism (five items, e.g., “I think the most beneficial thing about helping others is that will give me a better image”), compliance (two items, e.g., “I won't hesitate when others ask me for help”), emotion (four items, e.g., “The greatest sense of accomplishment for me is to comfort those who are in great pain”), and urgency (three items, e.g., “I'm willing to give help to those in distress or in urgent need”). The participants were rated on a 5-point Likert-type scale, with higher total scores representing a higher tendency to engage in PSB. The Cronbach's alpha was 0.874.

#### Social Support

The Perceived Social Support Scale (PSSS) developed by Zimet et al. (1988) was used to assess the perceived SS from various sources of SS, such as family, friends, teachers, and others. The scale consists of 12 items categorized into three dimensions, support from family, friends and others, and each dimension have 4 items. The scale is using a 7-point Likert-type scale (1 = strongly disagree; 7 = strongly agree) and all items are scored in forward. The total scores for all items were taken, with higher scores representing a higher level of the individual's perceived SS. The Cronbach's alpha was 0.911.

#### Self-Esteem

The Self-Esteem Scale developed by Rosenberg (1965) was used for the survey, and the domestic version was translated and revised by Wang Xiangdong and others (Xia et al., 2017). There were 10 items on the scale, of which five (3, 5, 8, 9, 10) were scored in reverse (e.g., “Ultimately, I tend to feel that I'm a loser”), and all items were scored on a 4-point scale (1 = very disagree; 4 = very agree). Tian (2016) found that the expression of question 8 was not consistent with the national culture; to improve the reliability and validity of the scale, it should be deleted or scored positively. In this study, question 8 was scored positively. The total scores for all items were taken, with higher scores representing a higher level of SE. The Cronbach's alpha was 0.714.

### Procedure

Undergraduates from multiple universities agreed to participate this offline survey. Before filling out the questionnaires, the experimenter explained the significance of the survey, emphasizing that it was anonymous and there were no right or wrong answers, and asking the participants to answer according to their actual situation. They were informed that they had the right to withdraw from it at any time. Participants answered in the order of EIS, PSSS, PTM and SES and it took about 30 minutes for the participants to complete all the questionnaires.

### Data Analysis

The SPSS21.0 software and the SPSS PROCESS macro program were used for data processing. First, we computed descriptive statistics and conducted Pearson correlations. Second, after all the data were standardized, based on 5,000 bootstrap samples (Hayes and Scharkow, 2013), the mediating effect of SS was analyzed using the PROCESS macro (Model 4) developed by Hayes (2015). Third, based on 5,000 bootstrap samples (Hayes and Scharkow, 2013), we used the PROCESS macro (Model 15) to examine whether SE moderated this mediation process. The effects are significant when the confidence intervals exclude zero.

## Results

Because this study collected data through self-reporting methods, it was possible that there could be an issue with common method variance (CMV). To reduce this possible deviation, according to the suggestion by Zhou and Long (2004), in the data collection stage, the participants were told that the results would be kept anonymous and that some items were reverse coded (Zhou and Long, 2004). After the data collection was complete, Harman's one-factor test (Podsakoff et al., 2003) was used to detect CMV because it is the most widely used method and is sensitive under most conditions (Fuller et al., 2015). The result of Exploratory Factor Analysis (EFA) showed a total of 16 factors with eigenvalues greater than one, and the first factor to explain the variance accounted for 19.17%, which was less than the critical value of 40%. Consequently, there was no significant CMV in this study.

### Descriptive Statistics and Correlation Analysis

As shown in Table 1, EI was found to be positively correlated with SS and PSB. SS was found to be positively correlated with PSB. SE was found to be negatively correlated with EI and SS. Furthermore, SE was not found to be correlated with PSB.

**Table 1:** Means, standard deviations, and correlations among variables.

**Variable**	**1**	**2**	**3**	**4**
1 Emotional intelligence	1			
2 Social support	0.47^**^	1		
3 Self-esteem	−0.11^**^	−0.14^**^	1	
4 Prosocial behavior	0.54^**^	0.34^**^	0.06	1
M	3.60	5.05	2.56	3.30
SD	0.46	0.95	0.42	0.51

*N = 742*.

** *p < 0.01*.

### The Mediating Role of Social Support

**Figure 2:**
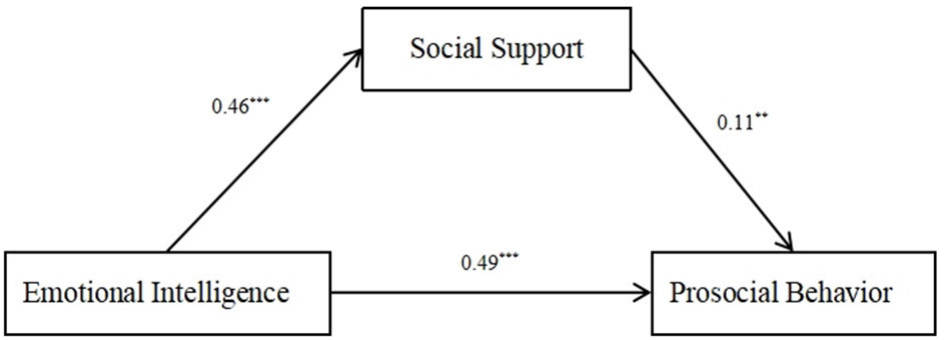
Path models examining the mediation role of social support between emotional intelligence and prosocial behavior. Unstandardized coefficients are presented. ***p < 0.001, **p < 0.01.

The PROCESS model 4 was used to examine the mediating role of SS between EI and PSB. Table 2 summarizes the results of the regression tests. EI was found to have a significant positive predictive effect on PSB (β = 0.54, SE = 0.04, 95% CI = [0.47, 0.61]). After SS was incorporated as mediating variable into the equation, the positive predictive effect of EI on PSB was still significant (β = 0.49, SE = 0.04, 95% CI = [0.41, 0.57]). The positive predictive effect of EI on SS was found to be significant (β = 0.47, SE = 0.03, 95% CI = [0.40, 0.54]), and SS was found to have a significant positive predictive effect on PSB (β = 0.11, SE = 0.04, 95% CI = [0.04, 0.18]). Thus, SS was found to play a partial mediating role between EI and PSB. The model is shown in Figure 2.

**Table 2:** Testing the mediation effect of social support between emotional intelligence and prosocial behavior.

**Predictors**	**Model 1 (Prosocial behavior)**				**Model 2 (Social support)**				**Model 3 (Prosocial behavior)**			
	**β**	**SE**	**t**	**95% bootstrap CI**	**β**	**SE**	**t**	**95% bootstrap CI**	**β**	**SE**	**t**	**95% bootstrap CI**
Emotional intelligence	0.54	0.04	14.47^***^	[0.47, 0.61]	0.47	0.03	13.60^***^	[0.40, 0.54]	0.49	0.04	11.75^***^	[0.41, 0.57]
Social support									0.11	0.04	2.99^**^	[0.04, 0.18]
*R^2^*	0.29				0.22				0.30			
*F*	209.44^***^				185.06^***^				115.14^***^			

*N = 742*.

** *p < 0.01*,

*** *p < 0.001*.

### The Moderating Effect of Self-Esteem

**Figure 3:**
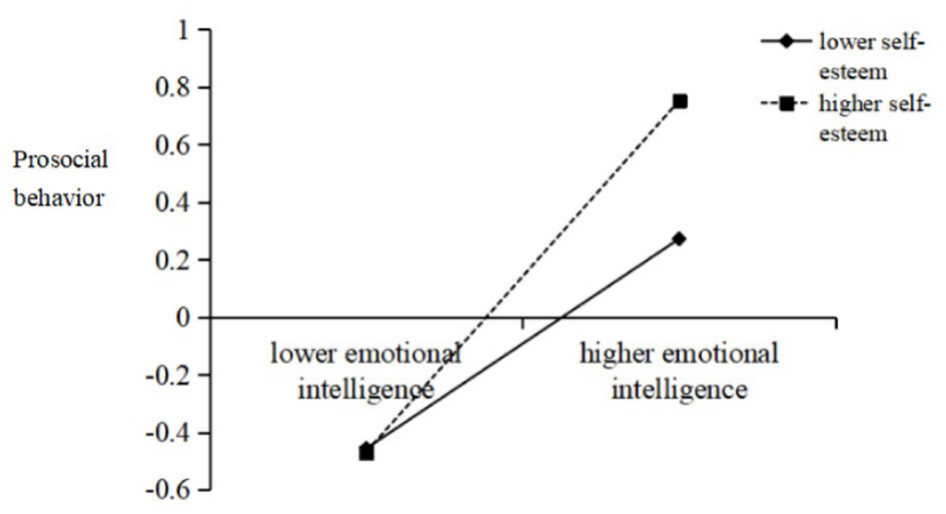
Interaction effect of emotional intelligence and self-esteem on prosocial behavior. High and low levels of emotional intelligence and self-esteem represent one standard deviation above and below the mean, respectively.

**Figure 4:**
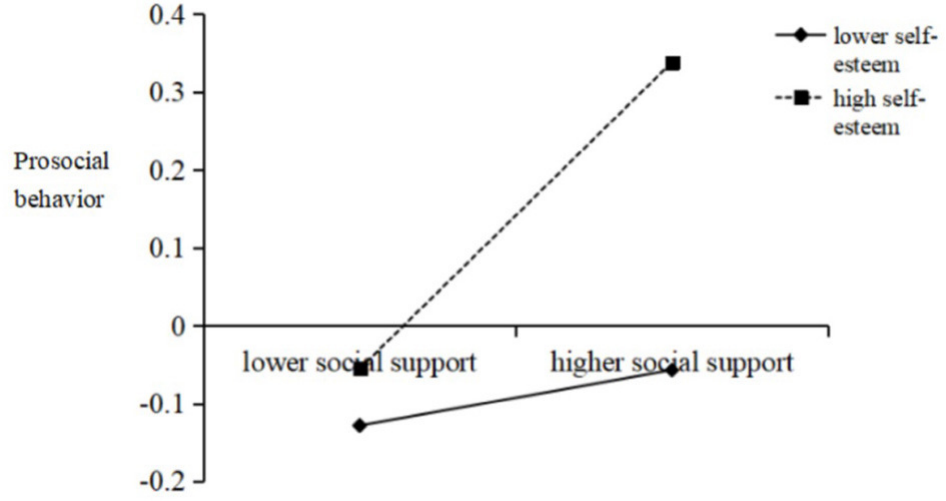
Interaction effect of social support and self-esteem on prosocial behavior. High and low levels of social support and self-esteem represent one standard deviation above and below the mean, respectively.

After identifying the indirect effect of SS on the relationship between EI and PSB, we investigated whether it was moderated by SE. The results demonstrated that the interaction of EI with SE significantly predicted PSB (β = 0.12, *p* < 0.01; see Model 2 of Table 3), and that the interaction of SS with SE significantly predicted PSB (β = 0.08, *p* < 0.05; see Model 2 of Table 3). Next, we plotted simple slopes, which predicted the relationship between EI and PSB as well as between SS and PSB, separately for high and low levels of SE. As presented in Figure 3, the slope of the association between EI and PSB was relatively weak for participants with high SE (β_highself−esteem_ = 0.61, *t* = 12.73, *p* < 0.001), whereas the slope was relatively strong when the SE of participants was low (β_lowself−esteem_ = 0.37, *t* = 7.60, *p* < 0.001). Additionally, as shown in Figure 4, the effect of SS on PSB was found to be significant for participants with high SE (β_highself−esteem_ = 0.20, *t* = 4.10, *p* < 0.001) but not for participants with low SE (β_lowself−esteem_ = 0.04, *t* = 0.74, *p*> *0*.05).

**Table 3:** Testing the moderated mediation effect of self-esteem.

**Predictors**	**Model 1 (Social support)**				**Model 2 (Prosocial behavior)**
	**β**	**SE**	**t**	**95% bootstrap CI**	**β**	**SE**	**t**	**95% bootstrap CI**
Emotional intelligence	0.47	0.03	13.60^***^	[0.40, 0.54]	0.49	0.04	12.65^***^	[0.41, 0.56]
Social support					0.12	0.04	3.20^**^	[0.04, 0.19]
Self-esteem					0.12	0.03	4.00^***^	[0.06, 0.17]
Emotional intelligence × self-esteem					0.12	0.04	3.21^**^	[0.05, 0.20]
Social support × self-esteem					0.08	0.04	2.28^*^	[0.01, 0.15]
*R^2^*	0.22				0.35			
*F*	185.06^***^				74.94^***^			

*N = 742*.

* *p < 0.05*,

** *p < 0.01*,

*** *p < 0.001*.

Then, we tested the conditional indirect effects of EI on PSB through SS. For participants with low SE, EI was found to have a lesser and indirect effect on PSB (β = 0.02, SE = 0.02, 95% CI = [−0.03, 0.06]), compared with those with high SE (β = 0.09, SE = 0.03, 95% CI = [0.04, 0.15]).

## Discussion

Based on PSB theory, EI theory, and existing research, the present study aimed to examine the link between EI and PSB. Overall, our findings supported our hypotheses. Generally, the results showed that EI is positively associated with PSB and that EI is indirectly associated with PSB through SS. This indirect effect is moderated by SE. Specifically, for individuals with high SE, SS can mediate the association between EI and PSB, whereas, for those with low SE, the mediating effect of SS was not significant.

### The Relationship Between Emotional Intelligence and Prosocial Behavior in College Students

The theory of PSB holds that the premise for PSB is that an individual must pay attention to the plight and needs of others, and EI is just a way of paying attention to the needs of others, experiencing the situations and emotions of others, and guiding one's behavior accordingly (Salovey and Mayer, 1989). In this study, we found that EI is positively associated with PSB. In line with previous research, our data suggested that individuals who have high EI generally engage in more PSB (Ciarrochi et al., 2002; Marc et al., 2004; Mavroveli and Sánchez-Ruiz, 2011; Kaltwasser et al., 2016; Martin-Raugh et al., 2016). EI contributes to individuals' socialization, so individuals with high EI can better perceive the needs of others and thus they can show more PSB (Mavroveli and Sánchez-Ruiz, 2011).

### Mediating Effect of Social Support

The results of this study also confirmed that SS partially mediates the relationship between EI and PSB. On the one hand, EI has a certain relationship with social factors such as interpersonal communication (Schutte et al., 2001), and individuals with high EI have better interpersonal relationships (Tang et al., 2015). SS reflects the closeness and quality of a person's connection with society, so individuals with high EI are more likely to get support from people around them. This study further validated the proposition that EI can significantly positively predict the SS of college students, which was consistent with previous findings (Kong et al., 2012; Ma and Wang, 2013; Martí-Vilar et al., 2019; Zhao et al., 2020). Salovey et al. (2002) also found that individuals with high EI have more positive interpersonal relationships and less conflict with others, and can get more emotional support from the SS system. When these individuals encounter negative emotions, they tend to draw more on SS. The findings of Zhao et al. (2020) clarify the underlying mechanism of EI, which can predict individual SS and PSB in a positive way. This is because college students with high EI have a strong ability to understand and infer their own emotions or those of others, which promotes their having more intimate social networks, which help them to obtain good external SS (Ma and Wang, 2013). Moreover, they can perceive well, use and regulate their own emotions and those of others, and thus frequently experience more positive emotions and fewer negative emotions (Zhao et al., 2020).

On the other hand, individuals with high SS engage in more PSB. The results of this study showed that SS is significantly positively correlated with PSB, indicating that the more SS college students receive, the more obvious their tendency to engage in PSB is, as suggested by previous studies (Tian et al., 2016; Wouter et al., 2018; Li et al., 2019). This showed that SS is an important environmental factor for PSB in college students. Moreover, when individuals perceive themselves as having a good interpersonal environment and close organizational relationships, they will have a strong sense of belonging, which in turn promotes altruistic behavior (Twenge et al., 2007; Wei et al., 2017). This shows that positive SS provides a good environment for the generation and development of PSB (Guzman et al., 2013).

To sum up, the results of this study showed that high EI provides individuals with a better ability to interact with others. This ability promotes the improvement of college students' SS, meaning that they will obtain more benefits and have a stronger sense of belonging. Such individuals will be more willing to engage in PSB, such as sharing and helping. In the meantime, from the perspective of SS, the investigation of the mediating role of SS is not only helpful in understanding the mechanism through which EI affects PSB but also in understanding the factors that influence the formation of individual positive qualities, to better develop individual potential. The results of this study enriched the exploration of the antecedent variables of SS and verified the influence of SS on individual behavior among college students. In light of these results, EI must be regarded as an important stimulant factor that improves PSB. Meanwhile, the results of this study remind us that to increase the probability of college students engaging in PSB, on the one hand, we can cultivate individuals' EI to enhance their perception of the needs and emotions of others, and drive them to help others. On the other hand, the tendency of college students to engage in PSB can be improved by enhancing their perceived SS.

### Moderating Effect of Self-Esteem

Previous studies have found that personality variables have a deep impact on PSB. Among the Big Five personality traits, agreeableness, conscientiousness, and neuroticism are highly correlated with PSB and can positively predict PSB (Ashton et al., 1998). Based on PSB theory and social connection theory, the research proposed two moderated roles. The results showed that SE has a significant regulating effect on the direct effect of EI on PSB and between SS and PSB.

This study supported the moderating effect of SE on the direct effect between EI and PSB; that was, the direct effect of EI on PSB in college students was moderated by SE. This may be because both EI and SE are important personality variables that affect individuals' PSB (Qi and Liu, 2013; Xiao and Hou, 2017). When both are at high levels, individuals are more likely to recognize the feelings of others and judge whether they need help, thereby making them more likely to engage in PSB. For individuals with high SE, when they have high EI, they can perceive the emotions, feelings, and needs of others well. Their ability to use this information to guide their behavior and maintain a positive attitude toward things will, therefore, promote the influence of EI on PSB. However, for high-SE individuals, when they have low EI, they tend to adopt negative coping styles to avoid failure when facing stressful situations. Even if individuals believe in their own judgment, they may also engage in less PSB to avoid the risk of helping others. Therefore, the higher the EI of individuals with high SE, the greater the tendency to engage in PSB they have, and the lower the EI, the less their tendency to engage in PSB will be. However, low-SE individuals tend to be negative in their self-evaluation and to show withdrawal, a sense of inferiority, and a lack of self-confidence in communicating with others (Shi et al., 2017), so they are less likely to interact with others or perceive the needs of others, which makes it is difficult for them to engage in helping behavior.

SE has a significant regulating effect between SS and PSB. Specifically, for individuals with high SE, SS can significantly predict PSB, whereas, for individuals with low SE, SS has no significant predictive effect on PSB. This can be explained by the social gauge theory of SE. The level of SE influences the maintenance of a good relationship between individuals and others. Individuals with high SE evaluate themselves more objectively, and they seldom use anger, hostility, or aggression against others to maintain their SE (Li, 2016) and are, therefore, more willing to engage in PSB. Moreover, SS is an individual-centered system composed of individuals and the people around them, as well as the social interactions between individuals and these people (Zhu et al., 2016); individuals with high SE usually evaluate themselves positively and show self-confidence (Murrell et al., 2003), and the optimistic attitude changes the individual's SS system—that is to say, it changes the individual's interactions with others, and then promotes their tendency to engage in PSB.

In conclusion, based on PSB theory, EI theory, and social exchange theory, this study investigated the mediating and moderating effect of EI on PSB, examined the joint effect of internal and external factors on PSB, and supplemented our understanding of the ways in and conditions under which EI promotes PSB. The integrated effect can better reflect the joint interaction of various systems and describe the effect of EI on PSB in different situations.

### Limitations

Some limitations of this study should be acknowledged. First, the cross-sectional design didn't provide evidence of a causal relationship between EI, SS, and PSB. Our findings, along with previous research, suggested a mechanism through which these factors may be related; longitudinal research is needed to determine whether the direction of the correlations may differ from what is assumed in our theoretical model. Second, although self-reported surveys have shown good reliability, shared method variance may have inflated the relationships found between instruments. Hence, future studies would benefit from using other formats to generalize our findings. For example, we could measure the real SS from parents, teachers, peers, etc. Third, there was a large gap in the proportion of majors and grades of the participants in this study. Thus, future research can pay attention to the selection of the participants to make their composition more representative. Fourth, this study found the mediating role of SS and the moderated effect of SE, respectively, but the moderated effect between SS and PSB was not limited to SE. Therefore, the mechanism of other variables between SS and PSB needs to be further explored. Finally, social expectation wasn't used as a control variable, when measuring the PSB in this study. Social expectation is that individuals make self-evaluation in order to make themselves more suitable for society. Therefore, in the future, researcher could include social expectation as a control variable to eliminate its potential confound on the results.

## Conclusions

Our results had two important implications. From a theoretical perspective, the present findings extended prior research by showing that SS can be an explanatory factor of EI and PSB. From a practical perspective, according to our findings, the link between EI and PSB was mediated by SS and moderated by SE. It suggests that developing one's SS and SE is important for the development of PSB of college student.

In general, it is necessary to comprehensively consider the external and internal factors of individuals and design a reasonable intervention plan to improve college students' PSB. Our study provided theoretical and empirical support for having mental health education courses for college students. This research explored the indirect path of EI to PSB. Therefore, in future mental health education courses, teachers will not have to only focus on cultivating EI, but can also formulate interventions to improve individuals' SS and SE. The courses can enable them to manage and express emotions more reasonably, thereby promoting them to exhibit more PSB.

## Data Availability Statement

The raw data supporting the conclusions of this article will be made available by the authors, without undue reservation.

## Ethics Statement

The studies involving human participants were reviewed and approved by the Academic Ethics Committee of the College of Psychology of Northeast Normal University. The patients/participants provided their written informed consent to participate in this study.

## Author Contributions

HW wrote and modified the manuscript. SW and WW collected and collated the data. CW recruited the participants. All authors contributed to the article and approved the submitted version.

## Conflict of Interest

The authors declare that the research was conducted in the absence of any commercial or financial relationships that could be construed as a potential conflict of interest.

## Publisher's Note

All claims expressed in this article are solely those of the authors and do not necessarily represent those of their affiliated organizations, or those of the publisher, the editors and the reviewers. Any product that may be evaluated in this article, or claim that may be made by its manufacturer, is not guaranteed or endorsed by the publisher.
